# Slow, deep breathing intervention improved symptoms and altered rectal sensitivity in patients with constipation-predominant irritable bowel syndrome

**DOI:** 10.3389/fnins.2022.1034547

**Published:** 2022-11-04

**Authors:** Jie Liu, Chaolan Lv, Wei Wang, Yizhou Huang, Bo Wang, Jiashuang Tian, Chenyu Sun, Yue Yu

**Affiliations:** ^1^Department of Gastroenterology, The First Affiliated Hospital of USTC, Division of Life Sciences and Medicine, University of Science and Technology of China, Hefei, China; ^2^Department of Gastroenterology, Affiliated Anhui Provincial Hospital, Anhui Medical University, Hefei, China; ^3^Graduate School of Bengbu Medical College, Bengbu, China; ^4^AMITA Health Saint Joseph Hospital Chicago, Chicago, IL, United States

**Keywords:** irritable bowel syndrome, constipation, anorectal function, slow deep breathing, autonomic dysfunction

## Abstract

**Background and aim:**

Limited treatment options have been shown to alter the natural course of irritable bowel syndrome (IBS). Slow, deep breathing (SDB) is a common pain self-management intervention. This pilot study aimed to explore the impact of SDB on measures of autonomic and anorectal functions as well as patient-reported symptoms in constipation-predominant IBS (IBS-C).

**Methods:**

Eighty-five IBS-C patients were enrolled in this study and randomly assigned to the experimental group (Group A, *n* = 42) and the control group (Group B, *n* = 43). SDB was conducted at six breathing cycles per minute with an inhalation for 4 s and exhalation for 6 s at a ratio of 2:3 and repeated for 30 min during the intervention. All subjects underwent high-resolution anorectal manometry (HRAM) and completed the standardized IBS symptom severity system (IBS-SSS) questionnaire. Meanwhile, changes in stool consistency, weekly frequency of complete spontaneous bowel movements (CSBMs), and weekly frequency of spontaneous bowel movements (SBMs) were recorded. All IBS-C patients received electrocardiogram (ECG) recordings for heart rate variability (HRV) analysis at baseline, weeks 3, 6.

**Results:**

At baseline, no differences were found between Groups A and B. The IBS-SSS score and its five sub-scores of Group B patients were significantly higher at week 6 than those of Group A patients (all *p* < 0.001). Furthermore, compared with Group B patients, Group A patients had a significantly higher threshold volume for the first sensation (*p* < 0.001), desire to defecate (*p* = 0.017), and maximum tolerable volume (*p* = 0.018) at week 6 of the SDB treatment. We also noted significant improvements in stool consistency (*p* = 0.002), weekly SBM frequencies (*p* < 0.001), and weekly CSBM frequencies (*p* = 0.018) of Group A patients at week 6 when compared with Group B patients. Finally, the corrected high frequency (HF) of Group A patients was significantly higher than the HF of Group B patients at week 3 (*p* < 0.001) and at week 6 (*p* < 0.001). Likewise, patients in Group A had a significantly higher root mean square of the successive differences (RMSSD) than that of patients in Group B at week 3 (*p* < 0.001) and at week 6 (*p* < 0.001).

**Conclusion:**

We found that a 6-week SDB intervention improved symptoms and altered rectal sensation in IBS-C patients. Moreover, SDB enhanced vagal activity. These findings suggest that the effect of SDB on IBS-C may be due to mechanisms involving autonomic responses.

## Introduction

Constipation-predominant irritable bowel syndrome (IBS-C), as one of the most common disorders of gut-brain interaction (DGBI), is defined under the Rome IV Diagnostic Criteria as recurrent abdominal pain/discomfort accompanied by changes in defecation habits and/or stool frequency or form (Ford et al., 2017). Globally, it is estimated that more than 11.2% of the population has irritable bowel syndrome (IBS) symptoms, of which IBS-C accounts for nearly a third of all IBS cases (Lacy et al., 2015; Ford et al., 2020). A previous study reported that patients’ quality of life could be significantly decreased by IBS-C (Ballou et al., 2019). IBS-C is well known to be linked to dysfunction of autonomic nervous system, visceral hypersensitivity, intestinal bacterial overgrowth, and gut inflammation, etc. (Holtmann et al., 2016). To date, of the approved IBS treatments, few have been confirmed to be effective in improving IBS-C symptoms. Dietary and behavioral interventions, prebiotic and probiotic supplements, spasmolytic agents, osmotic and/or stimulant laxatives, fiber products, and neuromodulators are recognized as common empirically supported therapies for IBS-C (Camilleri, 2021). Some novel drugs, including linaclotide, have been examined in randomized placebo-controlled trials and verified in IBS-C patients, but no pharmacological agent has been shown to alter the natural course of IBS (Black et al., 2020). In addition, there is no consensus on the gold standard treatment for IBS-C. Moreover, a recent meta-analysis revealed that diarrhea or headache was significantly more common in the licensed drugs treatment group than in the placebo group in IBS-C trials (Barberio et al., 2021). Therefore, it is important that, as an emerging area of interest, complementary and alternative medicine (CAM) are adequately investigated to determine its efficacy as a treatment for IBS.

Notably, previous evidences based on the American College of Gastroenterology Task Force demonstrated that IBS symptoms can be alleviated with dynamic psychotherapy, hypnotherapy, and cognitive therapy (American College of Gastroenterology Task Force on Irritable Bowel Syndrome et al., 2009). Slow, deep breathing (SDB) is a self-management intervention that ranks as the second most practiced complementary and alternative health approach according to National Health Interview Survey data (Clarke et al., 2015). SDB is a common component of several non-pharmacological techniques such as relaxation, meditation, hypnotherapy and yoga, which have been applied as a CAM in treating chronic pain syndromes (Nahin et al., 2015). A systematic review of several experimental studies employing somatic pain models and clinical studies of the effects of SDB on chronic pain management established the hypoalgesic effects of SDB on relieving pain symptoms (Jafari et al., 2017). They proposed cognitive, emotional, and autonomic (e.g., increased vagal nerve activity) modulations as potential mechanisms of SDB-mediated pain relief (Jafari et al., 2017).

Vagal afferent signaling can be increased using different levels of SDB (Jafari et al., 2017). The stimulation of pulmonary stretch receptors by deep breathing result in afferent signaling through the vagus nerve, which enhances afferent inputs to the nucleus of the solitary tract in the brain stem. These receptors also synapse with ascending circuits terminating at subcortical and cortical levels (e.g., insular cortex and amygdala) and participate in sensory, emotional, and cognitive processing of signals (Mazzone and Undem, 2016). Meanwhile, this nucleus of the solitary tract project directly and/or indirectly into several brain areas, which play a key role in pain regulation (e.g., periaqueductal gray and locus coeruleus) (Bruehl and Chung, 2004). Thus, on this theoretical basis, several studies have found hypoalgesic and antinociceptive effects of baroreceptor and vagus nerve stimulation (Busch et al., 2013; Reyes del Paso et al., 2014).

As high sympathetic tone and impaired vagal pathway are implicated in the pathophysiology of IBS and constipation (Tanaka et al., 2008; Liu et al., 2022), it is reasonable to hypothesize that SDB is a potentially effective, complementary lifestyle management in patients with IBS. A previous study found that microvascular endothelial function of IBS patients improved following the SDB intervention (Katherine Jurek et al., 2022). Although these observed improvements were encouraging, up to now, no study has assessed the effectiveness of SDB on anorectal physiological tests for IBS-C patients. The purpose of this pilot study was to explore the impact of SDB on measures of autonomic and rectal sensation as well as patient-reported IBS-C symptoms.

## Materials and methods

### Patients

Outpatients (ages 18–65) who met the Rome IV diagnostic criteria (Drossman, 2016) for IBS-C were diagnosed by a gastroenterologist (Y Y) experienced in the diagnosis of DGBI. Subjects were recruited into the study at the Department of Gastroenterology, the First Affiliated Hospital of University of Science and Technology of China (USTC) between July 2019 and January 2022. The exclusion criteria were: (i) patients who practice SDB on a regular basis; (ii) patients with chronic obstructive pulmonary diseases; (iii) patients with a history of any cardiovascular disease, including a heart attack, stroke, myocardial infarction, coronary artery disease, angina, arrhythmias; (iv) patients with a body mass index (BMI) of < 18.5 or > 30 kg/m^2^; and (iv) patients who were taking drugs that could affect IBS-C symptoms, such as prebiotic and probiotic supplements, spasmolytic agents, laxatives, and neuromodulators. Subjects were required to refrain from strenuous exercise, avoid caffeine and alcohol for at least 12 h, and fast (for solid food) for 4 h before testing.

This study protocol was approved by the Ethics Committee of the First Affiliated Hospital of USTC (Registration No: 2022-RE-142) and registered in the Chinese Clinical Trial Registry (No. ChiCTR-2200060462). All participants signed a written informed consent before their inclusion into the research.

### Experimental protocol

This was a within-subjects experimental study. A total of 90 patients with IBS-C were randomly divided into two groups: Group A and Group B. However, five participants were lost to follow-up due to time difficulties, representing a dropout rate of 6.67% for group A and 4.44% for group B. Finally, 85 IBS-C patients completed this study, including 42 IBS-C patients in the SDB intervention (Group A) and 43 IBS-C patients in the control group (Group B).

**FIGURE 1:**
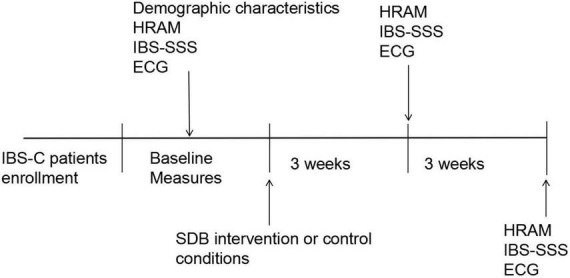
The study procedure. IBS-C, irritable bowel syndrome with constipation; HRAM, high-resolution anorectal manometry; ECG, electrocardiography; IBS-SSS, irritable bowel syndrome symptom severity system; SDB, slow, deep breathing.

All participants completed the standardized irritable bowel syndrome symptom severity system (IBS-SSS) questionnaire and pre- and post-SDB at weeks 3 and 6. Meanwhile, the stool consistency, weekly frequency of complete spontaneous bowel movements (CSBMs), and weekly frequency of spontaneous bowel movements (SBMs) of each patient were recorded. High-resolution anorectal manometry (HRAM) was performed for enrolled constipation-predominant IBS (IBS-C) patients, and all subjects received electrocardiogram (ECG) recording for time-domain heart rate variability (HRV) analysis at baseline, weeks 3, 6. The procedure of this study is depicted in Figure 1.

### Measurements

#### Slow, deep breathing intervention

This prospective study was a randomized control trial where subjects were not blinded to the study. Enrolled patients were randomly divided into Group A with a 6-week SDB intervention or Group B with control conditions. Randomization was performed using Randomizer.org while implementing a 1:1 allocation ratio. Patients in Group A were instructed to perform a 30-min SDB at least 5 days per week whereas the control group maintained their regular breathing. In detail, SDB was conducted at six breathing cycles per minute with an inhalation for 4 s and exhalation for 6 s at a ratio of 2:3 and repeated for 30 min during the intervention. Once baseline testing was completed, participants assigned to the SDB group performed all breathing sessions in the gastrointestinal motility room under the supervision of our research team. Practice logs were recorded to ensure that the experimental group met the practice requirements. Participants in both groups were instructed to maintain their regular diet and exercise regimens (Lee et al., 2021; Gholamrezaei et al., 2022).

#### High-resolution anorectal manometry

HRAM (MedKinetic, Ningbo, China) was performed for all IBS-C patients as described in our previous study (Liu et al., 2021), Briefly, 1–2 doses of glycerin enema was used to empty the rectum for bowel preparation 30–60 min before the HRAM examination. An eight-channel water-perfused anorectal manometric catheter (GAP-08A; Ningbo Maida Medical Device, Ningbo, China) was employed to measure the anal sphincter pressure and rectal sensation at a 1-cm interval. The device employs the technology of proprietary pressure transduction, which allows every pressure-sensor element to sense pressure over a 2.5-mm length in each of the 12 dispersed radially sectors. Patients were placed in the left lateral position and a catheter was inserted into the rectum after lubrication. The rectoanal inhibitory reflexes (RAIR) was then evaluated by inflating the balloon attached to the tip of the catheter with a hand-held syringe to a volume from 0 to 50 ml. The sensation test was assessed using a rectal balloon distended at a 5 ml interval until the subjects indicated the first sensation. Subsequently, we increased the volume of the balloon in progressive 5-ml increments until the subjects felt a sensation of the desire to defecate and the maximum tolerance. The outcomes for inducing these sensations depended on subject self-reports and the threshold volumes for these sensations were recorded (Lee and Bharucha, 2016).

#### Questionnaires

IBS-SSS is an internationally validated questionnaire for assessing overall IBS-like symptoms (Francis et al., 1997). The IBS-SSS score is a five subscore visual analog scales (VAS) instrument developed for measuring the intensity of IBS-like symptoms during the preceding 10 days. IBS-SSS consists of five subscores, including abdominal pain intensity and duration, stool frequency and consistency, abdominal distension, and interference with life in general. Each of the five subscores is rated on a 100-point Likert response scale, ranging from 0 (no symptom) to 100 (extremely severe symptoms). Thus, the highest possible total IBS-SSS score is 500 points. The higher the total score, the more severe the patient’s IBS-C symptoms. Meanwhile, the stool consistency [evaluated using the Bristol Stool Form Scale (BSFS)], weekly frequency of CSBMs, and weekly frequency of SBMs of each patient were also recorded during this study. The BSFS is a validated 7-point assessment that ranges from 1 [indicating separate, hard lumps, like nuts (hard to pass)] to 7 [indicating watery, no solid pieces (entirely liquid)] (Lewis and Heaton, 1997).

#### Assessment of autonomic functions

Autonomic functions were evaluated with spectral analysis of HRV time-domain as well as frequency-domain parameters. HRV signals were obtained using an ECG recording (ct-082, Hangzhou Baihui Electrocardiograms, China), whereas each subject’s HRV data was analyzed by monitoring R-R intervals with the HRV analysis software V.1.2.0.0 (Cardiotrak Holter system; Hangzhou Baihui Electrocardiograms, China). At baseline and control conditions, high-frequency (HF) power was used to reflect parasympathetic activity. Given the influence of SDB on the respiratory peak, we used the central frequency of respiration peak (CFRP) to correct HF band and eliminate the effect of SDB on respiratory peak shift, which in turn influenced our spectral analysis. The corrected HF area was chosen from CFRP*0.65 to 0.40 Hz (Chang et al., 2013; Li et al., 2018).

The Baevsky Index or Sympathetic Index (SI) was calculated to reflect sympathetic tone using the formula (Ali et al., 2021). The most frequent R-R interval was transformed into mode (Mo), expressed in seconds. A 50 ms bin width was used to calculate the amplitude of mode (AMo), expressed as a percentage of the total number of intervals measured. Variability was represented by MxDMn as the difference between longest (Mx) and shortest (Mn) R-R interval values, expressed in seconds. The SI was expressed as s^–2^.

Meanwhile, the following time domain HRV parameters were extracted based on the time between the individual R-peaks [the interval between R-peaks is defined as the normal to normal (NN) interval]: (1) standard deviation of NN-intervals (SDNN); (2) root mean square of the successive RR interval differences (RMSSD). In addition, previous study indicated that respiration had a limited impact on RMSSD, which could be used to reflect parasympathetic activity (Hill and Siebenbrock, 2009).

### Statistical analysis

All the statistical analyses were performed on SPSS V.19.0 software (IBM Corp, Armonk, NY). Continuous variables are given as mean ± standard deviation. Differences between the two groups were compared using a paired *t*-test for continuous variables and Chi-square test for discontinuous parameters. *P* < 0.05 signified statistical significance.

## Results

### Participants

A total of 90 IBS-C patients were enrolled in this study and randomly assigned into two groups: the experimental group (Group A) and the control group (Group B). However, five participants (three from group A and two from group B) were lost to follow-up. Of the 85 participants who completed the study, 59 were female and 26 were male, with a mean age of 46.79 ± 12.55 years. No statistical differences in age, gender, BMI, and the duration of IBS-C were observed among the three groups. These results are shown in Table 1.

**TABLE 1:** Demographic characteristics for the two study groups.

	Overall (*N* = 85)	Group A (*n* = 42)	Group B (*n* = 43)	t/χ^2^	*P*
**Gender**					
Male (n)	26	9	17	3.281	0.070
Female (n)	59	33	26		
Age (years)	46.79 ± 12.55	46.45 ± 11.70	47.12 ± 13.47	0.24	0.809
BMI (kg/m^2^)	23.34 ± 3.83	23.86 ± 3.98	22.83 ± 3.65	1.240	0.218
Duration of constipation (months)	34.81 ± 19.63	34.21 ± 10.30	35.40 ± 9.17	0.276	0.783

BMI, body mass index. Participants in group A were treated with slow, deep breathing and participants in group B were controls. No statistically significant difference was noted in age, gender, BMI, and the duration of constipation.

### Baseline data

Regarding rectal sensitivity, we found no significant differences between RAIR, first sensation, desire to defecate, and maximum tolerable volume of patients in Groups A and B (*p* = 0.176, *p* = 0.391, *p* = 0.133, and *p* = 0.073, respectively). Regarding IBS-C symptoms, we noted no difference in IBS-SSS between patients in Group A and B (*p* = 0.568). Regarding autonomic functions, we found no significant difference between patients in Groups A and B in HF (*p* = 0.667), SI (*p* = 0.121), SDNN (*p* = 0.852), and RMSSD (*p* = 0.435). Meanwhile, BSFS scores (*p* = 0.138), weekly CSBM frequencies (*p* = 0.669), and weekly SBM frequencies (*p* = 0.801) were comparable between the patients in the two groups. These results are summarized in Table 2. In addition, we considered the ranges mentioned in the study by Sun et al. (2014) as the reference. The volumes for the first sensation of 15 patients were below the normal range (20∼90 ml), and five patients were above the normal range. The volumes for the desire to defecate in 12 patients were below the normal range (50∼170 ml), and six patients were beyond the normal range. The volumes for the urge to defecate in eight patients were below the normal range (80∼220 ml), and four patients were beyond the normal range. The maximum tolerable volumes of 14 patients were below the normal range (120∼280 ml), and seven were above the normal range.

**TABLE 2:** Baseline characteristics for the two study groups.

	Overall (*N* = 85)	Group A (*n* = 42)	Group B (*n* = 43)	*T*	*P*
**Rectal sensitivity**					
RAIR (ml)	23.47 ± 5.23	23.92 ± 5.24	22.44 ± 4.80	1.364	0.176
First sensation (ml)	24.47 ± 5.72	23.93 ± 6.00	25.00 ± 5.46	0.862	0.391
Desire of defecation (ml)	51.18 ± 9.99	49.52 ± 9.03	52.79 ± 10.71	1.519	0.133
Urge to defecate (ml)	104.24 ± 10.51	103.45 ± 11.07	105.00 ± 10.00	0.677	0.500
Maximum tolerable volume (ml)	227.76 ± 45.50	218.81 ± 42.27	236.51 ± 47.30	1.818	0.073
**Heart rate variability (HRV)**					
HF (ms^2^)	135.01 ± 38.74	134.60 ± 37.58	135.42 ± 39.81	0.432	0.667
SI (s^–2^)	38.89 ± 7.18	40.12 ± 8.50	37.70 ± 5.44	1.567	0.121
SDNN (ms)	106.99 ± 18.64	106.81 ± 18.53	107.16 ± 18.83	0.187	0.852
RMSSD (ms)	26.45 ± 7.12	25.83 ± 6.24	27.05 ± 7.91	0.784	0.435
**IBS-C symptoms**					
IBS-SSS	284.00 ± 63.27	280.00 ± 47.52	287.91 ± 75.96	0.574	0.568
BSFS scores	1.64 ± 0.48	1.71 ± 0.46	1.56 ± 0.50	1.497	0.138
SBM frequencies (per week)	1.33 ± 0.59	1.36 ± 0.58	1.30 ± 0.60	0.430	0.669
CSBM frequencies (per week)	1.01 ± 0.42	1.00 ± 0.44	1.02 ± 0.41	0.252	0.801

RAIR, rectoanal inhibitory reflexes; HF, high frequency; SI, Baevsky Index or Sympathetic Index; SDNN, standard deviation of normal to normal intervals; RMSSD, root mean square of the successive differences; BSFS, Bristol Stool Form Scale; CSBM, complete spontaneous bowel movements; SBM, spontaneous bowel movements.

### Effects of slow, deep breathing on irritable bowel syndrome symptom severity system

**FIGURE 2:**
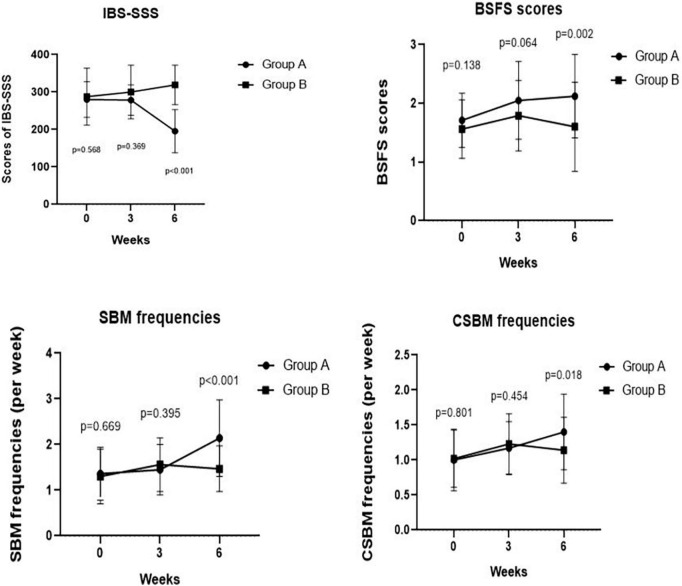
Comparing IBS-SSS score, BSFS score, weekly frequency of CSBMs, and weekly frequency of SBMs pre- and post-treatment at weeks 3, 6 between Groups A and B. We found a significant difference in IBS-SSS, BSFS score, weekly frequency of SBMs, weekly frequency of CSBMs post-treatment between Groups A and B at week 6 (*p* < 0.001, *p* = 0.002, *p* < 0.001, and *p* = 0.018, respectively).

As depicted in Figure 2, we observed no significant difference in IBS-SSS total score and five subscale scores at the baseline between Groups A and B (all *p* > 0.050). Meanwhile, we found no significant difference in IBS-SSS total score at week 3 between the two groups (288.57 ± 40.46 vs. 300.00 ± 71.58, *p* = 0.369), whereas the abdominal pain intensity score in Group B was significantly higher than that in Group A at week 3 (43.33 ± 14.59 vs. 56.28 ± 23.20, *p* = 0.003). Notably, patients in Group A had a significantly lower IBS-SSS total score and its five subscores compared to those in Group B after 6 weeks (all *p* < 0.001). These outcomes are shown in Table 3.

**TABLE 3:** Comparison of IBS symptom between the two study groups.

	3 weeks			6 weeks		
	Group A	Group B	*P*	Group A	Group B	*P*
Abdominal pain intensity	43.33 ± 14.59	56.28 ± 23.20	0.003	40.95 ± 12.46	64.19 ± 14.18	<0.001
Abdominal pain frequency	58.10 ± 11.53	59.07 ± 17.43	0.762	39.52 ± 15.61	64.65 ± 12.97	<0.001
Abdominal distension	60.00 ± 12.49	61.86 ± 17.36	0.573	35.71 ± 14.34	64.65 ± 15.02	<0.001
Dissatisfaction of bowel habit	60.48 ± 14.31	55.81 ± 16.07	0.056	40.00 ± 17.11	61.40 ± 17.67	<0.001
Interference on life in general	67.38 ± 23.48	70.00 ± 37.61	0.702	39.29 ± 19.68	64.19 ± 17.76	<0.001
IBS-SSS total score	288.57 ± 40.46	300.00 ± 71.58	0.369	195.48 ± 57.73	319.07 ± 52.45	<0.001

IBS, irritable bowel syndrome.

### Effects of slow, deep breathing on constipation symptoms

We observed no significant differences in the BSFS scores (2.05 ± 0.66 vs. 1.79 ± 0.60, *p* = 0.064), weekly SBM frequencies (1.45 ± 0.55 times vs. 1.56 ± 0.59 times, *p* = 0.395), and weekly CSBM frequencies (1.17 ± 0.38 times vs. 1.23 ± 0.43 times, *p* = 0.454) at week 3 between Groups A and B. However, we found significant improvements in BSFS scores (2.12 ± 0.71 vs. 1.60 ± 0.76, *p* = 0.002), weekly SBM frequencies (2.14 ± 0.84 times vs. 1.47 ± 0.50 times, *p* < 0.001), and weekly CSBM frequencies (1.40 ± 0.54 times vs. 1.14 ± 0.47 times, *p* = 0.018) of Group A patients compared with those of Group B patients at week 6 (Figure 2).

### Effects of slow, deep breathing on rectal sensitivity

**FIGURE 3:**
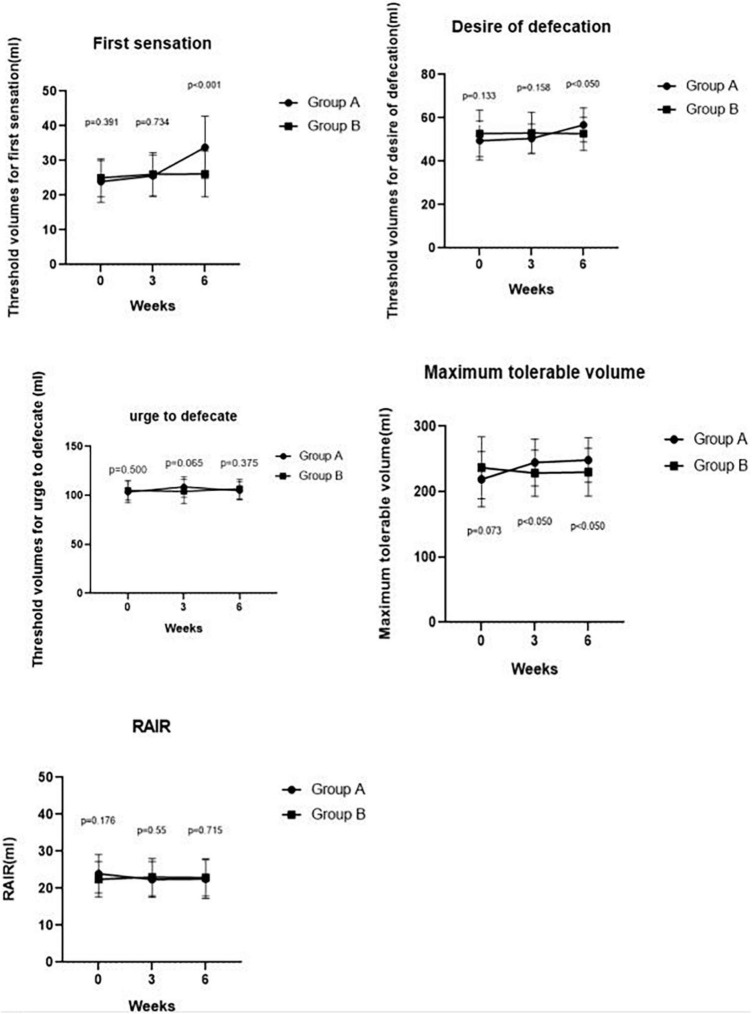
Comparing RAIR, threshold volumes for the first sensation, the desire to defecate, urge to defecate and maximum tolerable volume pre-treatment and post-treatment at weeks 3, 6 between Groups A and B. We found a significant difference in the maximum tolerable volume between Groups A and B at week 3 (*p* < 0.050). We also found significant differences in the first sensation, desire to defecate, and maximum tolerable volume between Groups A and Group B at week 6 (*p* < 0.001, *p* < 0.050, and *p* < 0.050, respectively).

Maximum tolerable volume was significantly higher in Group A than in Group B at week 3 (244.52 ± 35.90 ml vs. 228.37 ± 35.59 ml, *p* = 0.040). Compared with IBS-C patients in Group B, Group A patients had a significantly higher threshold volume for the first sensation at week 6 of SDB treatments (33.81 ± 8.96 ml vs. 26.16 ± 6.62 ml, *p* < 0.001), desire of defecation (56.79 ± 7.87 ml vs. 52.67 ± 7.66 ml, *p* = 0.017), and maximum tolerable volume (248.33 ± 34.07 ml vs. 229.77 ± 36.68 ml, *p* = 0.018). However, there was no difference in threshold volume for urge to defecate between Group A and Group B at week 3 (108.69 ± 10.42 ml vs. 104.07 ± 12.26 ml, *p* = 0.065) and at week 6 (104.76 ± 9.50 ml vs. 106.63 ± 9.80 ml, *p* = 0.375). Meanwhile, no difference was observed in threshold volume for the first sensation (25.60 ± 5.97 ml vs. 26.05 ± 6.22 ml, *p* = 0.734), desire of defecation (50.48 ± 6.70 ml vs. 53.02 ± 9.52 ml, *p* = 0.158) at week 3 between the two groups. In addition, there was no difference in RAIR between Group A and Group B after 3 weeks (22.38 ± 4.84 ml vs. 23.02 ± 5.02 ml, *p* = 0.550) and 6 weeks (22.50 ± 5.21 ml vs. 22.91 ± 5.03 ml, *p* = 0.715). These results are shown in Figure 3.

### Effects of slow, deep breathing on autonomic functions

**FIGURE 4:**
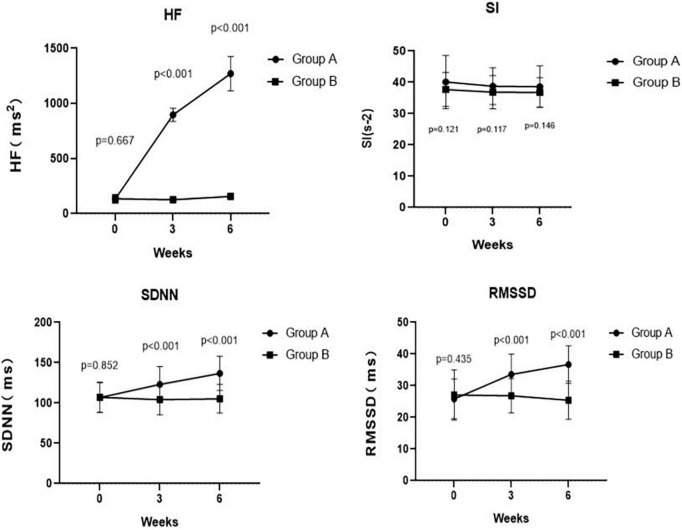
Comparing traditional or corrected HF, SI, SDNN, and RMSSD pre- and post-treatment at weeks 3, 6 between Groups A and B. The corrected HF in Group A was significantly higher than the HF in Group B at week 3 (*p* < 0.001) and at week 6 (*p* < 0.001). There was no significant difference in SI between Group A and Group B at week 3 (*p* = 0.117) and at week 6 (*p* = 0.146). At weeks 3, 6, the SDNN in Group A was significantly higher than that in Group B (*p* < 0.001 and *p* < 0.001, respectively). Meanwhile, at weeks 3, 6, the RMSSD in Group A was significantly higher than that in Group B (*p* < 0.001 and *p* < 0.001, respectively).

As summarized in Table 2, we found no differences in the HF (*p* = 0.667), SI (*p* = 0.121), SDNN (*p* = 0.852), and RMSSD (*p* = 0.435) between Group A and Group B at the baseline. Notably, the corrected HF in Group A was significantly higher than the HF in Group B at week 3 (897.19 ± 60.02 ms^2^ vs. 126.72 ± 16.62 ms^2^, *p* < 0.001) and at week 6 (1270.31 ± 155.61 ms^2^ vs. 157.26 ± 14.35 ms^2^, *p* < 0.001). However, there was no difference in SI between Group A and Group B at week 3 (38.76 ± 5.88 s^–2^ vs. 36.84 ± 5.32 s^–2^, *p* = 0.117) and at week 6 (38.59 ± 6.66 s^–2^ vs. 36.77 ± 4.68 s^–2^, *p* = 0.146). Meanwhile, the SDNN in Group A was significantly higher than that in Group B at week 3 (123.07 ± 22.03 ms vs. 104.23 ± 18.90 ms, *p* < 0.001) and at week 6 (136.83 ± 21.24 ms vs. 105.33 ± 17.70 ms, *p* < 0.001). Likewise, patients in Group A had a significantly higher RMSSD than that of patients in Group B at week 3 (33.55 ± 6.39 ms vs. 26.81 ± 5.40 ms, *p* < 0.001) and at week 6 (36.67 ± 5.94 ms vs. 25.40 ± 6.01 ms, *p* < 0.001). These results are shown in Figure 4.

## Discussion

The results of the current study demonstrated that a 6-week SDB intervention improved symptoms and altered rectal sensation in IBS-C patients. Meanwhile, patients receiving SDB intervention reported a significant improvement in stool consistency and the frequency of bowel movements. Moreover, according to the time-domain and frequency-domain analysis of HRV, SDB enhanced vagal activity in IBS-C. These findings suggest that SDB improved IBS-C symptoms, which may be mediated via enhancement of vagal activity. Notably, the relationship between SDB and its impact on rectal sensation remains largely unknown and scarcely studied in IBS-C patients. Results from this current study support the improved symptoms of a 6-week SDB intervention in IBS-C patients and suggest an increased threshold volume for the first sensation, desire of defecation, and maximum tolerable volume after 6 weeks of SDB intervention compared with control condition. To our knowledge, this is the first clinical trial study to demonstrate the impact of a SDB intervention on rectal sensitivity in patients with IBS-C.

In recent years, as pharmacological agents proven to be effective for the treatment of IBS-C are limited and have potential adverse effects, there has been considerable attention on lifestyle modifications and non-pharmacological interventions for IBS. As a result, complementary measures are frequently recommended but evidence for their effectiveness and adverse effects is scarce (American College of Gastroenterology Task Force on Irritable Bowel Syndrome et al., 2009; Black et al., 2020). This preliminary study shows that SDB is effective for self-management of IBS-C. In our study, of patients receiving SDB intervention exhibited significantly improved IBS-C symptoms after 6 weeks compared with control condition. It could be that SDB is more feasible than other lifestyle managements like yoga, meditation or traditional exercise. For example, SDB can be practiced from various locations with less time commitment and minimal physical effort. Notably, the relationship between SDB and improved IBS symptoms in IBS-C patients may be multi-factorial, and the underlying mechanisms need further investigation. Katherine Jurek et al. (2022) found that IBS symptom severity was unaltered after 4 weeks of SDB intervention. However, our study found that IBS-SSS was ameliorated significantly after 6 weeks of SDB treatment. This difference could be attributed to different durations of treatment used in the two studies. However, our outcome of improved IBS symptoms is similar to studies by Silva and Motta (2013) and Zivkovic et al. (2017). Specifically, Silva and Motta (2013) found that the use of breathing exercises increased defecation frequency after 6 weeks for constipation patients but fecal incontinence remained unchanged, concluding that SDB may be a useful treatment for constipation. Similarly, Zivkovic et al. (2017) demonstrated that significant improvement in defecation frequency was experienced after diaphragmatic breathing exercises treatment in children with chronic constipation and fecal incontinence. The authors concluded that breathing exercises could be considered effective complementary therapy for bowel dysfunction. Meanwhile, emerging evidence suggest a positive effect of breath training on gastroesophageal reflux disease (GERD) symptoms, indicating that inspiratory muscle training could train crural fibers, positively influencing the antireflux barrier (Casale et al., 2016). However, it is worth mentioning that the outcome of our present study regarding IBS symptoms was represented only by changes in subjective data. Therefore, the possible mechanism of SDB still needs to be further explored.

Imbalance in autonomic system has been considered a pathophysiological factor for development of IBS. Our previous study (Yu et al., 2019) found that deep breathing training is effective for GERD patients with increasing lower esophageal sphincter pressure and decreasing gastroesophageal acid reflux, which may be mediated by increasing vagal activity. In a randomized controlled trial investigating the effects of SDB on HRV of healthy adults, Lee et al. (2021) found that healthy adults receiving SDB intervention had higher values of alpha to high-beta wave and higher HF value than control group, indicating a significant change in autonomic function between SDB intervention group and control group. However, SDB can induce respiratory peak shifting, and the effect of respiration on HRV is displayed as a respiratory peak, which is located in the HF band. Thus, without the simultaneous analysis of the respiration rate, the changes in HF power should not be regarded as definitive evidence of changes in autonomic balance. To correct the effect of a slow breathing rate on respiratory peak shift, which in turn influences the spectral analysis, we referenced the enhanced method of Li et al. (2018) and used CFRP at 0.65 Hz to correct the conjunction between HF bands of HRV. Our corrected spectral analysis showed that SDB enhanced vagal activity, which is similar to the results obtained by Chang et al. (2013). Interestingly, no difference was observed in sympathetic activity, which was represented by SI. Meanwhile, to avoid the influence of SDB on HF, the time domain HRV parameters, including SDNN and RMSSD, were extracted based on the time between the individual R-peaks. The outcomes of time-domain analysis in this study shown that SDB increased the RMSSD and indicated that SDB might enhance vagal activity, which is similar to the results obtained by Rovsing et al. (2021).

A recent study by Gholamrezaei et al. (2022) also observed that SDB could decrease visceral pain intensity. Botha et al. (2015) reported that the development of acid-induced esophageal hypersensitivity could be prevented by deep breathing. Visceral hypersensitivity has been confirmed as playing a key role in the pathophysiology of functional gastrointestinal disorders, such as non-cardiac chest pain, heartburn, and IBS (Simrén et al., 2018). Notably, visceral hypersensitivity is regarded as the most important pathophysiological mechanism for the development of IBS. It has been found that IBS patients are more likely to report lower volumes or pressures of first sensation of rectal pain than healthy adults (Whitehead et al., 1990; Bradette et al., 1994). A previous study found no difference in rectal compliance between IBS patients and control subjects and significantly higher sensory threshold of volumes for rectosigmoid discomfort and pain in healthy controls than in IBS patients, indicating the role of visceral hypersensitivity in IBS (Whitehead et al., 1990). Moreover, the phenomenon of hypersensitivity in IBS patients is not only limited to the rectum and colon, but it is also associated with the whole digestive tract, suggesting that alteration of visceral sensation may present as a pan-intestinal phenomenon. Trimble et al. (1995) reported that lower rectal sensory thresholds in IBS patients than in controls and lower sensory threshold volumes for both first sensation and pain evoked by balloon distension of the esophagus. In the current study, we found an increase in maximum tolerable volume to anorectal balloon distension in IBS-C patients after 3 weeks of SDB treatment. Moreover, a higher threshold of volumes for first sensation, desire of defecation, and maximum tolerable volume were observed in IBS-C patients after 6 weeks of SDB treatment compared with the control group. Overall, we detected altered rectal hypersensitivity in constipated IBS patients following SDB, but the effect of SDB on IBS-C warrants further investigation.

### Limitations

Some potential shortcomings need to be noted in this study. First, the values of rectal sensory thresholds tested using HRAM were subjectively recorded through patients’ self-reports. Therefore, the difference in the sensory threshold may be statistically significant but certainly not clinically significant as we analyzed no subgroup of patients. Second, sensory test in the current study was performed using HRAM with water perfusion, whereas most recent studies were implemented using solid state catheters. Although no differences at rest between the two types of catheters were reported in previous studies, greater sensitivity to rapid pressure changes was observed in solid-state catheters compared with water perfusion, which may lead to different results (Liem et al., 2012; Rasijeff et al., 2017). Finally, the sample size in this study was quite small and therefore large-scale multicenter studies are warranted in the future to explore this relationship. The relationship between SDB and its impact on visceral hypersensitivity in IBS-C also needs further verification.

## Conclusion

Overall, we explored the association between SDB and its impact on IBS-C symptoms and rectal sensation in patients with IBS-C. We found improved symptoms and altered rectal sensitivity after a 6-week SDB intervention for patients with IBS-C. Moreover, the effect of SDB on IBS-C symptoms might be mediated by increased vagal modulation.

## Data availability statement

The raw data supporting the conclusions of this article will be made available by the authors, without undue reservation.

## Ethics statement

The studies involving human participants were reviewed and approved by Ethics Committee of the First Affiliated Hospital of USTC (Registration No: 2022-RE-142). The patients/participants provided their written informed consent to participate in this study.

## Author contributions

YY planned the study and revised the manuscript critically. JL, CL, WW, YH, BW, JT, and YY performed the SDB and HRAM. JL and CL collected and interpreted the data. JL and CS drafted the manuscript. All authors read and approved the final manuscript.
